# Long-term follow-up of patients with congenital diaphragmatic hernia

**DOI:** 10.1136/wjps-2023-000758

**Published:** 2024-04-09

**Authors:** Nicole Cimbak, Terry L Buchmiller

**Affiliations:** Department of Pediatric Surgery, Boston Children's Hospital, Boston, MA, USA

**Keywords:** Congenital Abnormalities, Patient Outcome Assessment, Pediatrics

## Abstract

Neonates with congenital diaphragmatic hernia encounter a number of surgical and medical morbidities that persist into adulthood. As mortality improves for this population, these survivors warrant specialized follow-up for their unique disease-specific morbidities. Multidisciplinary congenital diaphragmatic hernia clinics are best positioned to address these complex long-term morbidities, provide long-term research outcomes, and help inform standardization of best practices in this cohort of patients. This review outlines long-term morbidities experienced by congenital diaphragmatic hernia survivors that can be addressed in a comprehensive follow-up clinic.

## Introduction

**Figure 1:**
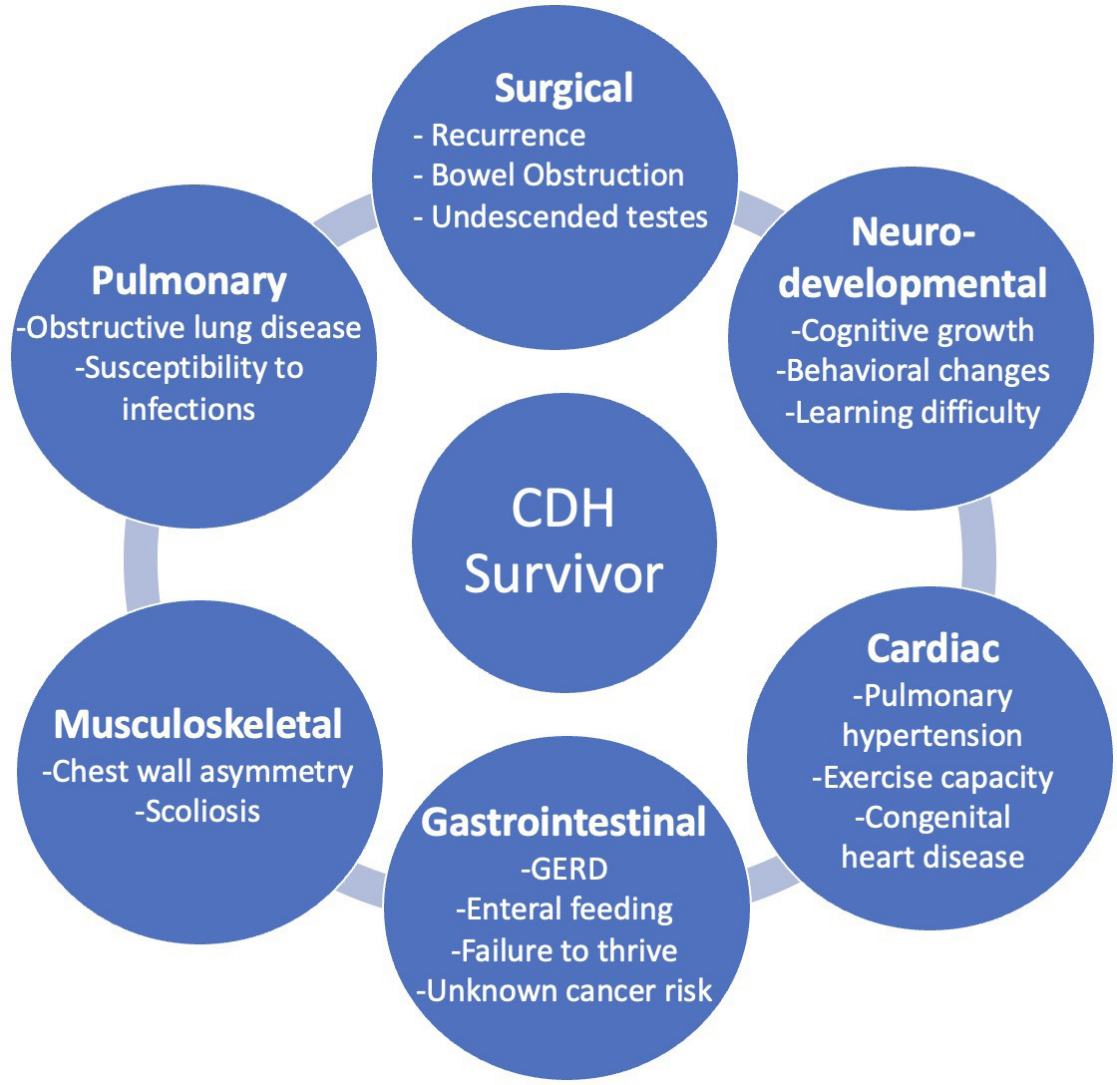
Multisystem disease morbidity profile of congenital diaphragmatic hernia (CDH) survivor. GERD, gastroesophageal reflux disease.

Overall survival in neonates born with congenital diaphragmatic hernia (CDH) has improved over the last 30 years.^1^ Contributing factors include advances in ventilation strategies, pulmonary hypertension (PH) therapies, standardized postnatal management protocols, extracorporeal membrane oxygenation (ECMO) utilization, and referral to high-volume specialized centers. With improved mortality, the growing number of CDH survivors often has complex multisystem comorbidities that require long-term management (figure 1).^2–9^ Dedicated pediatric practitioners have responded to this need with the formation of long-term multidisciplinary clinics along with research initiatives seeking to understand and manage long-term disease-specific morbidity in patients with CDH. This review will outline the importance of long-term CDH clinics and system-specific advances and recommendations for long-term follow-up care.

## Significance of long-term CDH clinics

Given the variation in CDH severity and pathophysiology, research collaboration in CDH outcomes forms the foundation for evidence-based management including the identification of predictors of survival to discharge from index hospitalization. The CDH Study Group (CDHSG) was founded in 1995 as a voluntary international data repository to address this need.^10^ While the CDHSG provides outcome data from index hospitalization, institution-based CDH clinics provide the majority of research on long-term outcomes in CDH neonates.

The first multidisciplinary long-term clinic was formed at Boston Children’s Hospital in 1990 by Wilson and colleagues. In 2008, the American Academy of Pediatrics (AAP) Section on Surgery published a report outlining recommended multidisciplinary follow-up (table 1).^11^ Similarly, the CDH EURO Consortium published guidelines in 2018 emphasizing multidisciplinary CDH postnatal care based on standardized clinical assessment and management plans.^12^ Center-specific protocols are not widely available but a review published in 2014 identifies commonalities to clinic structure including presence of a pediatric surgeon, pulmonologist, cardiologist, gastroenterologist and developmental pediatrician.^2^ Diagnostic studies completed at these visits vary but include a chest radiograph (CXR), echocardiogram (ECHO), neurodevelopmental assessment, and hearing screening. Other age-specific tests are also performed such as ventilation perfusion (V/Q) scans, pulmonary function tests (PFTs), and altitude testing.^13–16^

**Table 1:** Recommended follow-up schedule and diagnostic testing for CDH survivors

	Patient age at clinic visit			
	**Discharge to 1 year**	**Ages 1–2 years**	**Ages 2–5** **years**	**Ages 6–21 years**
Appointment schedule	Every 3 months	Every 6 months	Annually	Every 2–3 years
History and physical examination—includes scoliosis and chest wall deformity examination				
Chest X-ray				
V/Q scan				*
PFTs			5 years	As indicated
Echocardiogram		As indicated	As indicated	As indicated
Audiology			3 years	6 years
Upper GI study or pH probe	As indicated based on symptoms			
Cardiopulmonary exercise testing				As indicated
Neurodevelopmental assessment†	As indicated based on screening			
Brain imaging (MRI/CT)	ECMO survivors			
Altitude testing evaluation	Pending travel screen			

The shading indicates whether the intervention is recommended. Green signifies recommended and yellow signifies as indicated per patient.

*V/Q scan repeated if perfusion or ventilation to affected lung is <30%.

†Consideration of formal testing with Bayley III or IV or WPPSI-IV.

CDH, congenital diaphragmatic hernia; ECMO, extracorporeal membrane oxygenation; GI, gastrointestinal; PFT, pulmonary function test; V/Q, ventilation perfusion; WPPSI, Wechsler Preschool and Primary Scale of Intelligence.

Organizing a large number of specialty providers, diagnostic testing, and specialized resources is not always feasible. In a hospital setting without a centralized CDH clinic, families reported that time and distance to individual appointments were significant barriers to seeking follow-up care. Families also expressed interest in a CDH multidisciplinary clinic.^17^ Forming family-centered multidisciplinary clinics offloads the medical burden for families and broadens the catchment area where CDH survivors can receive multidisciplinary care. Along with providing improved care for CDH survivors, multidisciplinary clinics can collect and interpret data from their centers over longer survival periods.

While there are benefits to the granular research from single-center long-term clinics, collaborative long-term registries provide strength to evidence-based recommendations for follow-up. Chiu and Ijsselstijn outlined the creation of a prospective longitudinal database for multidisciplinary clinics to aggregate data through the CDHSG which is now in progress.^5^ These robust longitudinal clinic data will ultimately provide information to inform standardized follow-up protocols and guidance to the adult and pediatric community regarding optimal long-term management of these complex CDH survivors.

## Cardiopulmonary sequelae

### Pulmonary hypertension

The underdevelopment of the fetal lung parenchyma in CDH can result in severe pulmonary hypoplasia and abnormal pulmonary vasculature. The subsequent postnatal PH secondary to pulmonary arterial muscularization and pulmonary vasculature remodeling is often a source of severe morbidity and mortality in CDH neonates.^18–21^ While the majority of PH associated with CDH in infancy resolves over time, there is a subset of patients who have persistent PH warranting continued follow-up.^22^ Identifying which patients require long-term follow-up for PH with ECHO, cardiac catheterization, and titration of PH therapies remains a topic of research efforts.

Published reports cite PH rates after hospital discharge between 8% and 38%.^23 24^ There have been retrospective and larger database studies examining predictive factors for persistent PH severity to identify higher risk cardiac patients after discharge.^22 25–29^ These risk factors include duration of mechanical ventilation, ECMO utilization, and nitric oxide use during index hospitalization.^23 25^ Because of the associated long-term morbidity, the American Heart Association and American Thoracic Society report class I evidence for management of CDH neonates, including long-term monitoring with a PH specialist and ECHO evaluation.^30^

CDH-associated PH is particularly refractory to medical therapy, often invoking off-label use of PH medications.^31 32^ There has been an expansion of pharmacological PH therapies in patients with CDH targeting multiple PH pathways.^25 31 33–35^ Results of pharmacological studies have been promising with minimal associated clinically significant adverse events in CDH survivors.^31 36^ As the use of these medications increases, it follows that more neonates may be discharged on oral PH medications. Given the off-label use of PH medications in the pediatric population, management should be led by pediatric PH specialists in the outpatient setting, preferably in a multidisciplinary CDH clinic.

### Cardiac function

PH can reduce pulmonary blood flow with subsequent adaptations to the exercise response that differ from healthy controls.^37^ Cardiac capacity can be evaluated using cardiopulmonary exercise testing (CPET) in the outpatient setting. Multiple studies have examined CPET in CDH survivors, with initial testing at ages 7–10 years, showing quantitatively impaired exercise tolerance marked by lower peak oxygen consumption and minute ventilation. Patients report higher rates of dyspnea, feelings of throat closing, and effort perception compared to controls.^29 37–40^ Patients with higher rates of baseline activity had improved maximum exercise capacity compared with sedentary patients.^40^ Targeted initiatives, such as exercise programs for CDH survivors, based on collected data and research could improve cardiorespiratory capabilities.

### Concomitant neonatal congenital heart disease

There is a special population of survivors with both CDH and congenital heart disease (CHD) with a broad range of disease complexity. CHD may range from simpler conditions such as atrial septal defects, ventricular septal defects, and tetralogy of Fallot to complex cardiac disease including double outlet right ventricle, hypoplastic left heart syndrome and single ventricle lesions.^41^ Historically, complex CHD and CDH neonates were not typically offered ECMO, which affected survival in these complex neonates. Although CDH/CHD infants continue to have worse survival compared with CDH-only counterparts, improvements in management, including the availability of ECMO for appropriate patients, have led to survivors into adulthood.^42^ This special cohort requires close follow-up with congenital cardiac outpatient specialists in addition to CDH specialists.

### Lung function and development

Pulmonary function in CDH survivors can be quantified with PFTs, V/Q scans and chest tomography (CT). PFTs show significantly lower forced expiratory volume in 1 s (FEV_1_), forced vital capacity (FVC), and FEV_1_/FVC in CDH survivors.^15 16 38 43^ Factors predictive of decreased lung function included liver position within the chest, patch repair, ECMO utilization, and duration of mechanical ventilation.^8 16 44^ Alterations in PFTs persist into adulthood and can worsen over time.^15 45 46^ There is variability in the responsiveness of pulmonary function to bronchodilator therapy.^38 45 46^ Given the complexity of airway disease in patients with CDH, standardized evaluation by a pediatric pulmonologist is warranted to ensure appropriate diagnostic testing and medical management to optimize outcome.

Postnatal lung growth in patients with CDH is altered with decreased airway generation and radial alveolar counts in the ipsilateral lung accompanied by pulmonary muscularization in the contralateral lung.^19^ This remodeling is demonstrated by changes in V/Q imaging with lower rates (30–40% V/Q rates) correlating with increased pulmonary morbidity in survivors.^47^ Ipsilateral V/Q mismatch worsened over time in a long-term CDH neonatal population.^14^ As CDH-associated pulmonary morbidity worsens with age in a subset of patients, it is imperative to have longitudinal follow-up to identify these at-risk individuals who may benefit from tailored pulmonary therapies and rehabilitation. There is a small subset of patients whose severe pulmonary morbidity requires prolonged ventilatory support with tracheostomy placement. Factors predictive of tracheostomy include major cardiac anomalies, larger defect size, ECMO use, and intrathoracic liver.^48^ Patients with tracheostomies certainly mandate specialized pulmonary follow-up for ventilator weaning and if possible, future decannulation.

### Pulmonary protection

Given the pulmonary morbidity in patients with CDH, ensuring appropriate prophylaxis against respiratory infections, particularly influenza, COVID-19, and respiratory syncytial virus (RSV), is crucial.^49^ In a study of 201 patients with CDH, CDH neonates had a fourfold increased risk of hospitalization secondary to RSV compared with normal-risk infants. The role of palivizumab in preventing RSV in CDH survivors has recently been evaluated showing that CDH neonates benefit from vaccination.^50^ In July 2023, the monoclonal antibody, nirsevimab, was approved by the US Food and Drug Administration to protect newborns against RSV.^51^ The Advisory Committee on Immunization Practices and the Centers for Disease Control and Prevention in the USA unanimously approved the administration of nirsevimab for protection against RSV in neonates.^52^

CDH-associated pulmonary hypoplasia and chronic lung disease predispose patients to hypoxic events. Families should be counseled about air travel or prolonged time at higher altitudes. While there is not currently a standardization of altitude testing for patients with CDH, the British Thoracic Society recommends altitude testing for neonates less than 1 year old with a history of lung disease or any child who required supplemental oxygen in the prior 6 months.^53^ In one multidisciplinary CDH clinic, high altitude is simulated by providing a fraction of inspired oxygen of 0.15 via non-rebreather mask while oxygen saturation levels are monitored. Only 30% of patients who underwent high altitude testing were able to maintain oxygen saturations greater than 90% on initial attempt.^13^ Patients who do not pass altitude testing may benefit from supplemental oxygen use while at increased altitude levels. Additionally, CDH survivors should be counseled on the importance of avoiding smoking, vaping and secondary environmental exposures as incurred damage to already structurally altered lung parenchyma could lead to worsening pulmonary function.

### Interventions to address severe V/Q mismatch

**Figure 2:**
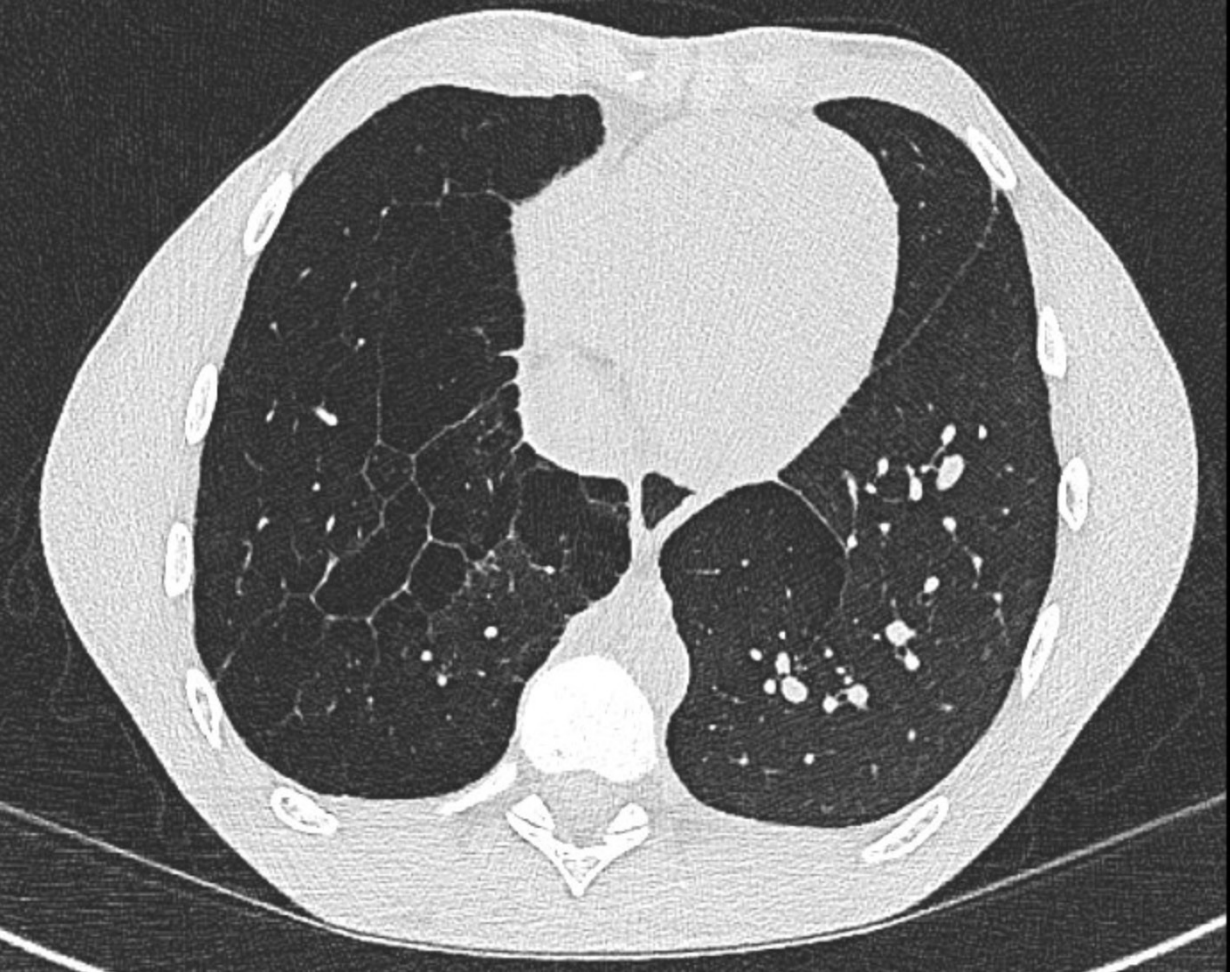
Severe air trapping and bullae in congenital diaphragmatic hernia (CDH) survivor.

Changes in perfusion and ventilation that worsen over time are associated with poor functional status.^14^ A small subset may also have air trapping with the development of emphysematous bullae in both the ipsilateral and contralateral lungs on CXR and CT imaging (figure 2).^54–57^ In adults with chronic obstructive pulmonary disease or emphysema, research is ongoing on how to manage severely damaged lung and large pulmonary bullae including use of endobronchial stents or valves and lung volume reduction surgery.^58–66^

## Gastrointestinal and nutritional sequelae

CDH survivors often have gastrointestinal and nutritional morbidities including growth impairment, gastroesophageal reflux disease (GERD), esophagitis, functional esophageal abnormalities, hypermetabolic states, and oral aversion.^67–70^ The occasional need for fluid restriction in the setting of pulmonary disease can contribute to the failure to reach growth goals.^4 71–73^ While gastrostomy placement theoretically allows for consistent caloric intake, it is not a reliable marker of achieving growth goals.^72 74 75^

GERD in CDH survivors is secondary to multiple factors including abnormal positioning of the gastroesophageal junction, impaired diaphragm crura function, intrathoracic stomach, liver position, and patch repair.^67–69 76–79^ GERD prevalence varies considerably in long-term clinic literature, ranging from 30% to 80%.^4 6 69 80 81^ High rates of silent esophagitis are reported, along with rare cases of Barrett’s esophagus and one case of esophageal adenocarcinoma.^70 82^ Unfortunately, despite this high risk of silent esophagitis, no reliable GERD predictive factors after age 6 years are identified.^67^

Most patients are discharged on acid suppression therapy for the treatment or prophylaxis of reflux, but the duration of treatment is undefined. Length of treatment can be based on symptom questionnaires or invasive testing.^83^ Understanding which patients would benefit from acid suppression warrants continuing investigation, as acid suppression therapy is not without its own side effects which can include alterations to bone healing and growth, depressed immunological function of the gastrointestinal and respiratory systems, and alterations in the gastrointestinal microbiome.^84^

Even more challenging is determining the role of fundoplication to improve growth, prevent respiratory infections and manage esophagitis.^78 79 85 86^ Prospective evaluation of concomitant fundoplication at time of CDH repair did not show a significant reduction in GERD symptoms in patients who underwent fundoplication compared with controls. Similarly, no difference in growth parameters was achieved at 2 years of age when comparing neonates who underwent fundoplication to controls.^85^ One of the largest studies from the French CDH Registry of over 700 neonates identified intrathoracic liver position, larger CDH defect size, prenatal diagnosis, and patch repair as predictive variables for fundoplication.^78^ As an alternative to fundoplication to manage GERD, postpyloric tube feeding has been used in some centers.

Close follow-up in a multidisciplinary CDH clinic with a gastroenterologist and a nutritionist is necessary as GERD symptoms and growth failure often persist throughout childhood. Ongoing failure to thrive has multiple adverse consequences including impact on cognition. It is important to identify these high-risk children who would benefit from nutrition-focused interventions such as home visits and family education.^72 87–89^

## Musculoskeletal changes

Musculoskeletal anomalies, including scoliosis and pectus deformities, occur in 20–48% of patients with CDH, much higher than the background incidence.^90–92^ Surgical approach is not associated with scoliosis rates, supporting the theory that scoliosis in CDH is congenital and not acquired.^93–96^ Patients with CDH require screening for scoliosis into adulthood and those with scoliosis benefit from early referral for specialized orthopedic interventions including bracing and potential surgery. Reported chest wall deformities include pectus excavatum (12–57%), pectus carinatum (2–13%) and chest wall asymmetry (49%).^43 92 95 97^ As with scoliosis deformities, CDH repair with primary closure, patch or muscle flap does not seem to be related to rates of chest wall deformities.^90 93 95 98 99^

Defect size does appear to be related to the incidence of chest wall deformities and scoliosis.^90 93^ Altered in utero development of musculoskeletal structures and the postnatal operative repair of CDH are both likely contributors to the presence and degree of these reported musculoskeletal anomalies. Discerning these prenatal and postnatal predictive factors will require review of patients with long-term CDH in multidisciplinary clinics to inform clinical decisions.

## Neurodevelopment

Neurodevelopmental morbidity is a significant sequela affecting multiple neurological domains: fine and gross motor skills, auditory ability, visuospatial perception, cognition, and language.^5 8 100–107^ Postnatally, neonates with critical illness have increased rates of neurodevelopment deficits that persist into adulthood.^108–111^ CDH neonates who use ECMO have impaired rates of verbal memory, working memory, visuospatial capabilities, cognitive processing and motor skills.^106 112^ MRI imaging in CDH survivors and ECMO survivors of other neonatal diseases shows alterations in the limbic system and white matter microstructure which correlated with neurological clinical sequelae in school-age children.^108^ Predictors of worse neurodevelopmental outcomes are related to disease severity: size of defect, ECMO utilization, patch repair, intrathoracic liver position and prolonged oxygen requirement.^102 113 114^ Delays in motor performance can be compounded by the inability to participate in physical exercise due to severe cardiopulmonary compromise.^100^

The AAP Section on Surgery recommends neurodevelopmental screening starting at 9–12 months and then annually until 5 years of age.^11^ Neurodevelopmental assessment tools are challenging to implement in a young population as the responses often rely on parent-reported outcomes and the training of the provider completing the assessment.^115–118^

Neurodevelopment beyond 5 years of age warrants continued evaluation as deficits can persist into school-age children and even adulthood.^103 105^ Schiller and colleagues have completed multiple studies on neurodevelopmental outcomes in CDH survivors. Their work supports the theory that neonates with a neurological insult may have deficits that become apparent with older age when higher neurocognitive processing is needed.^119 120^ CDH survivors have increased rates of learning disability, attention deficit hyperactivity disorder, and developmental disability compared with matched controls.^103 121 122^ Standardization of neurological assessment until adolescent survivors is supported as patients may benefit from additional academic support.

Interventions, such as cognitive-based repetition, show promise for neurodevelopment. A randomized controlled trial evaluating the impact of standardized and repetitive cognitive training in a group of CDH survivors aged 8–12 years showed improved verbal working memory and visuospatial memory after intervention. Improvements in working memory did not persist past the 1-year time point, possibly due to the cessation of the intervention.^119^ These types of cognitive training programs could be of benefit to this long-term population and be organized through multidisciplinary clinics.

Sensorineural hearing loss (SNHL) is an additional contributor to neurodevelopmental sequelae in 7–56%.^107 123 124^ Predictors of SNHL include duration of aminoglycoside treatment, loop diuretic therapy, inhaled nitric oxide, longer duration of mechanical ventilation, and use of high-frequency oscillation.^107 123 125^ Auditory evaluation prior to discharge with methodical screening thereafter is important to identify those at risk who could benefit from intervention.^4^ The AAP recommends at least one diagnostic audiology assessment by 24–30 months of age followed by developmentally appropriate audiological screening.^126^ ECMO utilization is also a significant risk factor for hearing loss, and this population should undergo enhanced surveillance screening.^127^

## Surgical needs

Patients often undergo additional surgical interventions after CDH repair such as feeding tube insertion, fundoplication, pectus excavatum repair, recurrent CDH repair, or operations for bowel obstruction. CDH recurrence rates vary considerably ranging from 3% to 20% with potential under-reporting due to lack of standardized radiological follow-up in many centers.^68 128–130^ The majority of recurrences, including those that may not undergo surgical intervention, occur within the first year of life. In one study, 35% of recurrences were identified on routine follow-up imaging in a multidisciplinary clinic.^128^ Monitoring for CDH recurrence and obstruction is particularly important as they can have life-threatening consequences.^4 75 79 128 131 132^

Risk factors for CDH recurrence include larger CDHSG defect size and patch repair.^133 134^ Meta-analysis showed a threefold higher risk of recurrence in CDH neonates who underwent thoracoscopic repair versus open repair.^135^ The ability to follow CDHSG patients longitudinally with prospective data collection will be of key importance to understanding the long-term recurrence rates in patients who undergo minimally invasive repair.

Bowel obstruction has been reported in up to 20% of survivors and can be attributed to adhesions, volvulus, or CDH recurrence.^68 128 132^ Patients with CDH have abnormal fixation of the bowel secondary to herniation into the chest which may contribute to the higher rates of bowel obstruction.^68 132^ One study showed that obstructive complications were higher in neonates with malrotation and non-fixed bowel documented on initial CDH repair. Documenting the rotational status during the initial CDH operations may help in predicting future risk. Additionally, concurrent Ladd procedure with the index CDH repair dependent on the clinical stability of the patient was shown to be protective against future small bowel obstructions in one study by Heiwegen and colleagues.^132 136^ Given the increased risk of obstructive pathology in neonates who undergo CDH repair regardless of repair type, a posterior-anterior and lateral chest radiograph is supported at every visit to evaluate for recurrence as well as patient/ family education regarding signs and symptoms of bowel obstruction.

Finally, there is a higher prevalence (18%) of undescended testes in males with CDH compared with the generalized population, and most commonly occur on the ipsilateral side as CDH defect. This is theorized to be secondary to decreased antenatal intra-abdominal pressure and possibly the absence of diaphragmatic tissue near the urogenital ridge leading to impaired testicular descent.^137 138^ Testicular position needs to be documented on physical examination, with surgical intervention undertaken according to established guidelines.

## Special populations

Fetal endoscopic tracheal occlusion (FETO) is a maternal/fetal intervention aimed towards improving the degree of pulmonary hypoplasia with minimally invasive techniques.^139^ Long-term outcomes in neonates who underwent FETO therapy are evolving. One study of 32 FETO neonates showed similar morbidity profiles though FETO survivors had higher rates of pulmonary morbidity at 2 years (oxygen, bronchodilator use) even after adjusting for disease severity.^140^ Sferra *et al* reported similar findings with 58% of FETO survivors requiring bronchodilator therapy or supplemental oxygen compared with non-FETO CDH survivors.^141^ FETO survivors are likely to have tracheomegaly which has not been found to have significant clinical impact.^142 143^ These long-term studies confirm that neonates who underwent FETO intervention have favorable long-term survival rates and similar morbidity that should be managed in specialized long-term CDH clinics.

## Psychosocial impact on patient and family

Even with significant morbidity associated with CDH, most survivors report similar quality of life compared with controls in multiple domains including physical well-being, psychosocial well-being, autonomy, and feeling of belonging in a school or community.^144–147^ Michel and colleagues reported the largest contributors to decreased quality of life were poor peer/social support and lack of autonomy.^148^

Primary caregivers bear much of the emotional and financial burden of caring for CDH children in the long term. Parents of CDH neonates report use of medical equipment utilization (62%), home health services (18%), and special education services (28%).^146 149^ 45% of families had to change their previous employment status to care for a child with CDH. Families with less financial stability have worse emotional well-being scores on quality of life surveys.^150^ Support systems for parents of children with CDH have grown to include social media outlets and discussion boards. Interviewed parents commented on the anxiety of caring for a CDH child, particularly immediately after discharge, and reported the importance of having reliable follow-up, emergency plans, and a point of contact for issues as they arise.^151^ Multidisciplinary clinics can provide this reliable follow-up with regular telemedicine and in-person clinic visits.

## Transitioning to adult care

Long-term follow-up for patients with CDH should extend into adulthood as many alterations in cardiopulmonary physiology, nutrition, neurodevelopment and musculoskeletal development persist beyond adolescence.^152^ Attention to appropriate transition of care models has increased for a variety of congenital anomalies. There are multiple barriers to transitioning to adult care including lack of patient interest and paucity of adult provider expertise.^153^ Significant planning is required to transition these patients to multiple adult providers.

Long-term clinics have begun publishing on important patient benchmarks for transitioning care, including patient and adult provider disease-specific education about relevant surgical anatomy, yearly readiness assessments, and collaborative initiatives between pediatric and adult providers.^154–156^ The AAP, American College of Physicians and American Academy of Family Physicians published a detailed guide for the transition of care citing six core elements vital for the transition process.^157^ These six tenets outline specific guidelines and considerations when transitioning care, including autonomy of decision-making in adult care, information privacy, and patient readiness questionnaires.^157^ Patients must become their own advocates, which is often a new role. Adequate education and preparation should begin early in long-term clinics to facilitate smooth transitions.

## Research opportunities

This review has presented follow-up data from long-term clinics and larger multicenter analyses. One significant assumption of long-term clinic data analysis is that the patients who are followed in long-term CDH clinics represent the patient population that was originally discharged. In the long-term multidisciplinary clinic at Boston Children’s Hospital, unpublished data have shown a 30% clinic attrition rate. However, there is a similar disease severity profile of active patients compared with those who are lost to follow-up. We encourage long-term clinics to include information about attrition rates in their publications so that readers may better understand possible selection biases impacting the reported outcomes.

CDH neonates who survive into adulthood remain a target for research, particularly the manifestation of cardiopulmonary alterations with aging. Kraemer *et al* published outcomes in a CDH survivor population with a median age of 23 years. Peak oxygen consumption and O_2_ pulse were significantly lower in ECMO-treated CDH survivors during exercise function. Right ventricular systolic pressure was significantly elevated in ECMO-treated group compared with non-ECMO survivors.^29^ These authors elected to additionally complete a sex-based analysis which prompts the question of how cardiopulmonary function in female CDH survivors could be altered in pregnancy. No studies to date have been able to answer this question and it is certainly of importance to counseling in long-term CDH clinics.

We urge researchers to contribute to larger international CDH registry groups and to publish information on these relevant topics in CDH survivors. These research initiatives can lead to future evidence-based management practice guidelines.

## Conclusion

The emerging population of patients with CDH continues to survive into adulthood. While many are confronted by chronic health conditions that require management, the majority of survivors report high quality of life and many are living without any sequelae of their neonatal disease state. CDH multidisciplinary clinics have increased to meet the needs of this population and will continue to evolve the care for these highly complex patients. Longitudinal data from these long-term clinics will be crucial to inform our best practices and to work towards standardized transition of care models.

## Data Availability

No data are available.
